# Do Gender and Race/Ethnicity Influence Acute Myocardial Infarction Quality of Care in a Hospital with a Large Hispanic Patient and Provider Representation?

**DOI:** 10.1155/2013/975393

**Published:** 2013-12-30

**Authors:** Tomás Romero, Pablo Velez, Dale Glaser, Camila X. Romero

**Affiliations:** ^1^University of California, San Diego, Sharp Health Care, San Diego, USA; ^2^Sharp Chula Vista Medical Center, San Diego, California, USA; ^3^University of San Diego, California, USA; ^4^University of New Mexico, Albuquerque, New Mexico, USA

## Abstract

*Background*. Disparities in acute myocardial infarction (AMI) care for women and minorities have been extensively reported in United States but with limited information on Hispanics. *Methods*. Medical records of 287 (62%) Hispanic and 176 (38%) non-Hispanic white (NHW) patients and 245 women (53%) admitted with suspected AMI to a southern California nonprofit community hospital with a large Hispanic patient and provider representation were reviewed. Baseline characteristics, outcomes (mortality, CATH, PCI, CABG, and use of pertinent drug therapy), and medical insurance were analyzed according to gender, Hispanic and NHW race/ethnicity when AMI was confirmed. For categorical variables, 2 × 2 chi-square analysis was conducted. Odds ratio and 95% confidence interval for outcomes adjusted for gender, race/ethnicity, cardiovascular risk factors, and insurance were obtained. *Results*. Women and Hispanics had similar drug therapy, CATH, PCI, and mortality as men and NHW when AMI was confirmed (*n* = 387). Hispanics had less private insurance than NHW (31.4% versus 56.3%, *P* < 0.001); no significant differences were found according to gender. *Conclusions*. No differences in quality measures and outcomes were found for women and between Hispanic and NHW in AMI patients admitted to a facility with a large Hispanic representation. Disparities in medical insurance showed no influence on these findings.

## 1. Introduction

Gender and race/ethnic disparities have been often reported in United States with women and minority groups receiving less cardiac catheterization (CATH), thrombolytic therapy, percutaneous coronary interventions (PCI), coronary artery bypass graft (CABG) surgery, aspirin (ASA), beta-blockers, angiotensin converting enzyme inhibitors (ACEI)/angiotensin receptor blockers (ARB), statins, and referral to cardiac rehabilitation than men and Whites. These disparities have been documented extensively in women and African-Americans [1–13] but a paucity of current information exists in Hispanics, who have been frequently underrepresented in the pertinent literature. In general, most of the information in Hispanics has either been obtained more than 10 years ago [6, 14–16] and/or from hospitals with a proportionally very limited Hispanic patient representation (not beyond 1–5% of the data base analyzed) [6, 15–17]. In health care the interaction of socioeconomic factors with the cultural characteristics of patients and providers has a universally recognized importance [18, 19]. The objective of the present study is to provide insights in this issue analyzing the experience of a hospital with a large Hispanic representation.

## 2. Methods

Medical records of 607 consecutive patients admitted with a suspected acute myocardial infarction (AMI) between January 2004 and December 2006 to a southern California nonprofit community Hospital with on site tertiary level of cardiac care were reviewed.

Twenty-four percent of the registered nurses (RN) and technical staff, 32% of the admitting physicians to the medical floor and intensive care unit, 62% of the cardiologists, and 40% of the cardiac surgeons were Hispanics. Baseline information of age, gender, race/ethnicity, previously documented coronary heart disease (MI, previous coronary revascularization), cardiovascular risk factors (CVRF) (history of hypertension, diabetes, dyslipidemia, smoking, congestive heart failure, renal failure), and medical insurance were obtained from the medical records.

### 2.1. Definition of Variables

#### 2.1.1. Race and Ethnicity

They are Self-reported by the patients or direct members of the family on admission. 463 patients (245 women) had Hispanic (287, 62%) or Non-Hispanic White (176, 38%) ethnicity and were the subjects of the study. Approximately 85% of patients classified as Hispanics reported a Mexican ancestry or origin.

### 2.2. CVRF

Identified by data from the medical records provided by patients, family members, and/or previous admissions. History or evidence of use of medications for hypertension, diabetes, or lipid disorders was considered confirmatory of a risk factor. Smokers were considered those who were current smokers for the 12 months prior to their hospital admission. Creatinine values of 1.8 mg/% or greater, or hemodialysis, were considered indicative of renal failure.

Standard ER and inpatient diagnostic procedures (serial EKG, Troponin I, and CPK) according to the currently accepted diagnostic criteria were utilized to confirm an AM I [20]. Stress testing, CATH, and coronary revascularization procedures were performed as recommended by the consulting or attending cardiologist and patient's preference.

Referral to Cardiac Rehabilitation was provided to all in-patients with a confirmed AMI or patients subject to revascularization procedures, and included inpatient physical activities, dietary, and smoking cessation counseling.

Excluded were patients that presented to the ER with a cardiopulmonary arrest in progress, or those who were transferred to other facilities.

### 2.3. Outcomes Assessment

The following outcomes were measured: hospital mortality, all AMI (Non ST segment elevation MI + STEMI), stress testing, cardiac catheterization (CATH), percutaneous coronary interventions (PCI), coronary bypass graft surgery (CABG), and use (in hospital and at discharge) of ASA, beta-blockers, statins/antilipidemics, ACEI)/ARB, and clopidogrel for patients with a confirmed AMI. A composite drug index was calculated averaging the proportion of in-hospital and discharge use of these medications. Very few patients (*N* = 12) were treated with thrombolytic therapy and were not included in the data analysis.

### 2.4. Medical Insurance Characteristics

Medical insurance was analyzed considering the following groups.

(1) Private insurance, including all types of primary or supplemental insurances not financed by the government or state (i.e., private fee-for-service insurance, Health Maintenance Organizations).

(2) Nonprivate insurance, composed of all primary government and/or state financed insurance. These corresponded to Medical, Medicare/Medical, Community Health Center for Medicare & Medicaid Services (patients without insurance at the time of admission but considered eligible for Medical, or Medicare-Medical) and patients with no insurance and not eligible for government or state financed insurance.

### 2.5. Statistical Analysis

Between group comparisons for all patients with confirmed MI (*n* = 387) were performed for the following pair wise comparisons:

1. men versus women
2. Hispanic versus non-Hispanic White.

For the categorical variables 2 × 2 chi-square analysis was conducted. Besides the *P* value, for designated outcomes (death, CATH, PCI, CABG, and stress testing) the Odds Ratio (OR) and 95% confidence interval adjusted by multivariable logistic regression considering as predictors age, gender, Hispanic/non-Hispanic White ethnicity, CVRF, private/non private medical insurance were obtained. For the composite drug use index a Poisson regression was calculated for the same outcomes and predictors. For testing differences in proportions (specifically for the insurance analyses) a SPSS macro was downloaded that specifically furnishes tests of significance via the *z* test of difference in proportions [21].

## 3. Results

### 3.1. Baseline Characteristics

#### 3.1.1. Men versus Women

No differences were observed between men and women with respect to age, history of diabetes, hypertension, and lipid disorders in patients with a confirmed AMI. Previous MI, smoking, and history of CHF were more frequent in men (Table 1).

No differences were noted in the proportion of private or nonprivate insurance according to gender. However, women had more Medical (11.8 versus 6.0%, *P* = 0.025), Community Health insurance and other nonprivate insurance (8.6 versus 3.7%, *P* = 0.026) than men (data not shown in Tables).

#### 3.1.2. Hispanics versus Non-Hispanic Whites

Hispanics were younger than non-Hispanic whites and had more diabetes but similar BMI, an other CVRF (Table 1). In a subgroup analysis (data not included in Tables) Hispanic women reported less smoking than non-Hispanic White women (21.9% versus 37.5%, *P* = 0.026); no differences were found between their male counterparts (45.2 versus 37.1 respectively, *P* = 0.23).

Hispanics had more nonprivate (government and state financed insurance) than non-Hispanic whites (66.8% versus 43.0%, *P* < 0.001) (Table 1).

### 3.2. Outcomes

#### 3.2.1. Men versus Women

Men had a higher proportion of confirmed AMI and STEMI and received more CATH, PCI and CABG than women, but had similar mortality. Women, on the other hand, had more stress testing than men in patients admitted with a suspected AMI (Table 2). However, in patients with a confirmed AMI after multivariable logistic adjustment these gender differences disappear for cardiac catheterization and PCI but persisted for CABG (Tables 2 and 3).

#### 3.2.2. Hispanics versus Non-Hispanic Whites

After full adjustment for all predictors, Hispanics had similar mortality, CATH, PCI, CABG and composite drug use than non-Hispanic Whites. (Tables 3 and 4).

#### 3.2.3. Cardiovascular Risk Factors and Outcomes

After adjustment for all predictors, age and renal failure increased significantly mortality (Tables 3 and 4), diabetics received more PCI and CABG, and in contrast, patients with renal failure had less CATH, PCI, and a reduced composite drug use (Tables 3 and 4).

#### 3.2.4. Medical Insurance and Outcomes

No apparent relationship was documented between the type of medical insurance and the proportion of patients that received CATH, PCI, CABG, stress testing and composite drug use (Tables 3 and 4). Patients with private insurance showed a lower mortality after adjustment for all the predictors than those with non private insurance when an AMI was confirmed (OR 0.33, 95% CI 0.12–0.99, Table 3). No differences in age and CVRF were noted according to insurance status (data not included in tables).

## 4. Discussion

The key findings of our study, conducted in a facility with a large representation of Hispanic patients and providers may be summarized as follows: (1) no race/ethnic or gender disparities were found in AMI care, (2) use of diagnostic and therapeutic resources were consistent with current expected performance measures [22]. These results are in contrast with previous publications on this subject [6, 13, 15, 23, 24].

Although demographically different than many other facilities where Hispanics are admitted, the site of the study was similar in reflecting the usual care of AMI patients in United States. Our findings of higher rates of diabetes in Hispanics than in non-Hispanic whites and lower smoking prevalence in Hispanic women have been previously well documented [25, 26]. It was also not surprising the larger number of confirmed AMI in men after admission considering the higher proportion of cardiovascular risk factors they presented with, in comparison to women.

Two studies with large databases that required voluntary enrollment of hospitals for monitoring guidelines and performance measures (GWTG-CAD) [27] or were part of multicenter randomized management protocols (SYNERGY) showed no significant disparities of care in Hispanics. However, because of the characteristics of these studies their results likely do not reflect the reality of usual care in most US hospitals. A more recent publication noted higher AMI readmissions rates for Hispanics regardless of the hospital characteristics (Hispanic serving hospital were defined as those that cared for an average of 12.5% of Hispanic patients while 0.5% in the non-Hispanic serving hospital). A lower quality of care score for the Hispanic serving hospitals was noted, and these were in larger proportion for profit and teaching facilities compared to their counterparts [28].

In our study, Hispanics had significantly more nonprivate insurance (state or government subsidized medical insurances provided to low-income individuals or no insurance) than non-Hispanic whites, which are consistent with previous studies in minority groups in the USA [13, 16, 23, 24, 29]. Medical insurance, among other factors, has been considered a proxy for socio-economic status, and was suggested as a source of provider bias [29–31]. In contrast to previous published reports, differences in medical insurance showed no apparent influence in the use of invasive and revascularization procedures or drug therapy in our study [29, 30]. There were no differences in death rate between Hispanics and non-Hispanic whites, but a higher mortality was noted in patients with confirmed AMI who had nonprivate insurance after multivariable logistic adjustment. It is conceivable that socioeconomic determinants that were not measured in this study, such as education, income, and environmental characteristics could have been at play as it has been extensively reported in the lower socioeconomic strata [18, 19, 32, 33].

Multiple factors ranging from socioeconomic, patient and cultural characteristics, to provider bias and racial/ethnic stereotyping have been suggested to account for the reported disparities of care in women and minorities [6, 18, 29, 30]. Culturally competent health care systems have been proposed to improve disparities in care for minorities. Although there is published information suggesting their effectiveness in health care of minorities [34–37], there have been no studies regarding their impact in the quality of care and outcomes of Hispanics admitted with an AMI. Moreover, there is no current information focused on the quality of care received by Hispanic women presenting with an AMI.

**Figure 1:**
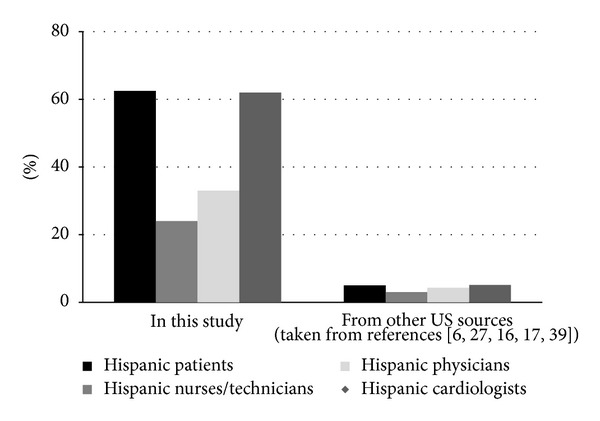
Proportion of Hispanic patients, nurses/technicians and physicians from the hospital in this study and from the database published from other US facilities and sources.

Hispanics are a culturally and racially heterogeneous group with different degrees of acculturation to the U.S. main stream. Data from the US Census Bureau indicate that 47.9% of Hispanic patients admitted to hospitals speak only Spanish or English “less than very well”, but not more than 7% of the civilian employed health care professionals (RN, physicians, technicians) of the U.S are Hispanic [38]. Although the current national US figures for hospital Hispanic health care professionals are unknown, that number is probably a reasonable approximation. Data from the Association of the American Medical Colleges indicates that 5.1% of the cardiologists graduated from USA medical schools were Hispanics in 2004 [39]. These figures are in striking contrast with the much larger proportion of Hispanic care providers involved in our study as illustrated in Figure 1. However, it is uncertain if acculturation and cultural competency indicators influenced these results because they were not directly evaluated in our study. It is also unclear if the services available at our site were determinant factors since the information published on this issue has been inconclusive [9, 13, 29, 40].

### 4.1. Limitations

First, a major limitation of this study is its small sample size and limited generalizability since it reflects the experience of a single hospital with a large proportion of Hispanic patients and providers. These characteristics different from most hospitals in the United States, may have minimized acculturation and access barriers for Hispanics and enhanced cultural competency of the site, factors that probably would have contributed to a larger impact on disparities. On the other hand, the site where the study was conducted is similar to many other facilities in United States since it was not enrolled in studies that could have influenced the management of patients, and therefore it is closer to the reality of usual care of many other hospitals. Second, the presence of cardiovascular risk factors was only determined by information provided by the patients and/or the family members, or by data from previous admissions. Finally, the door to balloon time for PCI procedures in patients presenting with STEMI was not measured, which is an important quality of care indicator in the management of AMI.

## 5. Conclusions

No disparities in the quality of care or outcomes related to gender, race/ethnicity, or medical insurance characteristics between Hispanics and non-Hispanic Whites admitted with a suspected AMI to a facility with a large Hispanic representation of patients and providers were found. These results may reflect a combination of factors that include the widespread implementation of current guidelines of care in U.S., the sociocultural features of a hospital with a large Hispanic representation of patients and providers and the characteristics of the on site services. Future studies including a larger sample of hospitals with a substantial Hispanic representation from various United States regions along with the assessment of indicators of acculturation and cultural competency may add more insights on this issue.

## Figures and Tables

**Table 1:** Baseline characteristics of patients admitted with acute myocardial infarction (AMI) according to gender and race ethnicity.

	Men		Women		*P* value	Hispanics		Non-Hispanic whites		*P*-value
	*N* (or M)	% (or SD)	*N* (or M)	% (or SD)		*N* (or M)	% (or SD)	*N* (or M)	% (or SD)	
Total (*n* = 387)	212	54.8	175	45.2		229	59.2	158	40.8	
Age	*68.25 *	*14.983 *	*67.72 *	*13.124 *	0.68	*66.85 *	*13.92 *	*69.87 *	*14.30 *	**0.026**
BMI (kg/m^2^)	*27.31 *	*4.70 *	*28.44 *	*6.79 *	0.043	*28.14 *	*7.38 *	*28.74 *	*11.05 *	0.669
Hx of diabetes	94	44.3	90	51.4	0.165	123	53.7	61	38.6	**0.003**
Hx hypertension	147	69.3	136	77.7	0.064	173	75.5	110	69.6	0.196
Hx of smoking	88	41.5	48	27.4	**0.004**	77	33.6	59	37.3	0.452
Hx dyslipidemia	115	54.2	108	61.7	0.139	135	59	88	55.7	0.524
Hx of previous MI	73	34.4	42	24	**0.025**	74	32.3	41	25.9	0.178
Hx coronary revasc.	48	22.5	35	20	0.529	50	21.8	33	20.9	0.823
Hx of renal failure	53	25.4	32	18.3	**0.112**	57	24.9	28	17.7	0.094
Hx of CVA	29	13.7	20	11.4	0.58	26	11.4	23	14.6	0.352
Hx of CHF	80	37.7	50	28.6	**0.050**	75	32.8	55	34.8	0.673
Private insurance	96	45.3	70	40	0.298	76	33.2	90	57	**<0.001**
non-private insurance	116	54.7	105	60.0	0.112	153	66.8	68	43.0	**<0.001**

BMI: Body mass index; Hx: history; MI: myocardial infarction; Revasc.: revascularization; CVA: cerebrovascular accident; CHF: congestive heart failure.

**Table 2:** Selected outcomes in patients admitted with a suspected (*n* = 463) and confirmed (*n* = 387) Acute Myocardial Infarction according to gender and race ethnicity.

	Men		Women		*P* value	Hispanics		Non-Hispanic whites		*P* value
	*N* (or M)	% (or SD)	*N* (or M)	% (or SD)		*N* (or M)	% (or SD)	*N* (or M)	% (or SD)	
Suspected AMI (*n* = 463)										
Death	16	7.3	10	4.1	0.129	15	5.2	11	6.3	0.22
Confirmed AMI	212	97.2	175	71.4	**<0.001**	229	79.8	158	89.8	**0.005**
Stress testing	10	4.6	54	22	**<0.001**	43	15.0	21	11.9	0.36
CATH	152	69.7	126	51.4	**<0.001**	171	59.6	107	60.8	0.79
PCI	80	36.7	54	22	**<0.001**	82	28.6	52	29.5	0.82
CABG	49	22.5	25	10.2	**<0.001**	50	17.4	24	13.6	0.28
Confirmed AMI (*n* = 387)										
Death	16	7.5	10	5.7	0.47	15	6.6	11	7	0.87
STEMI	77	36.3	33	18.9	**<0.001**	59	25.8	51	32.3	0.167
CATH	147	69.3	110	62.9	0.179	154	67.2	103	65.2	0.67
PCI	78	36.8	52	29.7	0.142	78	34.1	52	32.9	0.81
CABG	46	21.7	24	13.7	**0.042**	47	20.5	23	14.6	0.134
ASA (Hosp)	193	93.7	126	92.1	0.566	185	93.0	136	93.2	0.95
Beta blockers (Hosp)	182	88.3	119	85.6	0.46	175	87.9	126	86.3	0.65
ACEI/ARB (Hosp)	130	63.1	91	65.5	0.65	125	62.8	96	65.8	0.57
Statins/Antilipidemics (Hosp)	154	75.5	104	74.8	0.89	146	73.7	112	77.2	0.46
Clopidogrel (Hosp)	108	52.4	71	51.1	0.81	104	52.3	75	51.4	0.87
ASA (DC)	165	87.3	119	88.1	0.82	165	88.7	119	86.2	0.50
Beta Blockers (DC)	149	78.4	102	75.6	0.54	148	79.1	103	74.6	0.34
ACEI/ARB (DC)	94	50	74	55.2	0.36	95	51.6	73	52.9	0.82
Statins/Antilipidemics (DC)	135	71.4	96	71.1	0.95	135	72.6	96	69.6	0.55
Clopidogrel (DC)	100	53.2	67	49.6	0.53	95	51.1	72	52.6	0.79
Composite medications index	104	75.96	150	76.00	0.96	146	76.2	107	75.73	0.924

AMI: acute myocardial infarction; CATH: cardiac catheterization; PCI: percutaneous coronary intervention; CABG: coronary artery bypass graft; STEMI: ST segment elevation myocardial infarction; ASA: aspirin; Hosp: prescribed on admission; ACEI/ARB: angiotensin converting enzyme inhibitor/Angiotensin receptor blocker; DC: prescribed at discharge.

**Table 3:** Odds ratio for selected outcomes in Hispanic and non-Hispanic white patients with a confirmed AMI.

Predictors	Death		Cardiac cath		PCI		CABG	
	OR (95% CI)	*P* value	OR (95% CI)	*P* value	OR (95% CI)	*P* value	OR (95% CI)	*P* value
Age	**1.04** (1.00, 1.09)	**0.037**	**0.97** (0.95, 0.99)	**<0.001**	0.98 (0.97, 1.0)	0.059	0.99 (0.97, 1.01)	0.155
Men versus women	1.33 (0.55, 3.22)	0.53	1.39 (0.87, 2.21)	0.16	1.37 (0.87, 2.16)	0.19	1.77 (0.99, 3.16)	0.05
Hisp. versus non-Hispanic Whites	0.84 (0.34, 2.07)	0.71	1.04 (0.64, 1.67)	0.88	1.02 (0.64,1.63)	0.92	1.56 (0.86, 2.83)	0.14
Hx diabetes.	0.79 (0.32, 1.93)	0.60	1.06 (0.66, 1.71)	0.81	**1.54** (0.97, 2.46)	**0.049**	**2.26** (1.26, 4.07)	**0.006**
Hx hypertension.	1.38 (0.44, 4.40)	0.58	1.22 (0.71, 2.11)	0.47	0.71 (0.43, 1.18)	0.191	0.69 (0.37, 1.28)	0.24
Hx smoking	0.71 (0.26, 1.96)	0.51	1.55 (0.94, 2.57)	0.087	1.38 (0.86, 2.20)	0.179	0.62 (1.99, 0.48)	1.11
Renal failure	**3.03 **(1.25, 7.37)	**0.014**	**0.39** (0.22, 0.67)	**0.001**	**0.47** (0.26, 0.86)	**0.014**	0.72 (0.35, 1.47)	0.36
Hx Prev. MI	0.82 (0.29, 2.27)	0.70	0.81 (0.47, 1.40)	0.45	1.01 (0.60, 1.71)	0.96	1.43 (0.76, 2.70)	0.26
Hx Prev. Revasc.	1.66 (0.60, 4.63)	0.33	0.89 (0.49, 1.61)	0.69	1.22 (0.68, 2.17)	**0.51**	0.24 (0.09, 0.63)	**0.003**
Private versus nonprivate insurance	0.35 (0.12, 0.99)	**0.049**	0.66 (0.41, 1.07)	0.093	0.81 (0.51, 1.30)	0.39	1.27 (0.71, 2.26)	0.42

AMI: acute myocardial infarction; CATH: cardiac catheterization; PCI: percutaneous coronary intervention; CABG: coronary artery by-pass graft; Hisp.: Hispanic; Hx: History; Prev. MI: previous myocardial infarction; Prev. Revasc.: Previous coronary revascularization (PCI and/or CABG). *Outcomes adjusted for age, gender, race/ethnicity, history of diabetes, hypertension, smoking, renal failure, previous MI, and previous CABG/PCI.

**Table 4:** Events rates (Poisson regression) in Hispanic and non-Hispanic patients with a confirmed AMI for a composite drug use by multivariable logistic regression of the listed predictors.

Composite drug index	(All AMI *n* = 387)		Event R.	95% CI	
Predictors	*b*	*P*-value	
Age	−0.002	0.157	1.00	0.99	1.00
Men versus women	−0.022	0.66	0.98	0.89	1.07
Hispanic/non-Hispanic white	0.010	0.83	1.01	0.92	1.11
Hx diabetes	0.037	0.044	1.04	0.95	1.14
Hx hypertension	0.063	0.23	1.07	0.96	1.18
Hx smoking	−0.010	0.084	0.99	0.90	1.09
Renal failure	−0.173	**0.003**	**0.84**	0.75	0.94
Hx previous MI	−0.045	0.41	0.96	0.86	1.06
Hx previous revascularization	0.106	0.082	1.11	0.99	1.25
Private versus nonprivate insurance	0.041	0.38	1.04	0.95	1.14

AMI: acute myocardial infarction; Drug Index: proportion of in-hospital and discharge use of drugs (please see Methods for details); Hx: history; MI: myocardial infarction.

## Abbreviations

CATH: Cardiac catheterization
PCI: percutaneous coronary intervention (angioplasty and/or stent)
CABG: Coronary artery bypass graft
ACEI: Angiotensin converting enzyme inhibitor
ARB: Angiotensin receptor blocker
CVRF: Cardiovascular risk factor
NHW: White.

## References

[1] Cook NL (2010). Disparities in cardiovascular care: does a rising tide lift all boats?. *Circulation*.

[2] Ayanian JZ, Epstein AM (1991). Differences in the use of procedures between women and men hospitalized for coronary heart disease. *New England Journal of Medicine*.

[3] Pfeffer MA, Moye LA, Braunwald E (1991). Selection bias in the use of thrombolytic therapy in acute myocardial infarction. *Journal of the American Medical Association*.

[4] Thomas RJ (1996). National survey on gender differences in cardiac rehabilitation programs: patient characteristics and enrollment patterns. *Journal of Cardiopulmonary Rehabilitation*.

[5] Stone PH, Thompson B, Anderson HV (1996). Influence of race, sex, and age on management of unstable angina and non-Q-wave myocardial infarction: the TIMI III registry. *Journal of the American Medical Association*.

[6] Correa-de-Araujo R, Stevens B, Moy E, Nilasena D, Chesley F, McDermott K (2006). Gender differences across racial and ethnic groups in the quality of care for acute myocardial infarction and heart failure associated with comorbidities. *Women’s Health Issues*.

[7] (2009). *Disparities in Health Care Quality among Racial and Ethnic Minority Groups: Findings from the National Healthcare Quality and Disparities Reports, 2008*.

[8] Keil JE, Sutherland SE, Knapp RG, Lackland DT, Gazes PC, Tyroler HA (1993). Mortality rates and risk factors for coronary disease in black as compared with white men and women. *New England Journal of Medicine*.

[9] Whittle J, Conigliaro J, Good CB, Lofgren RP (1993). Racial differences in the use of invasive cardiovascular procedures in the Department of Veterans Affairs Medical System. *New England Journal of Medicine*.

[10] Taylor HA, Canto JG, Sanderson B, Rogers WJ, Hilbe J (1998). Management and outcomes for black patients with acute myocardial infarction in the reperfusion era. *American Journal of Cardiology*.

[11] Chen J, Rathore SS, Radford MJ, Wang Y, Krumholz HM (2001). Racial differences in the use of cardiac catheterization after acute myocardial infarction. *New England Journal of Medicine*.

[12] Spencer F, Scleparis G, Goldberg RJ, Yarzebski J, Lessard D, Gore JM (2001). Decade-long trends (1986 to 1997) in the medical treatment of patients with acute myocardial infarction: a community-wide perspective. *American Heart Journal*.

[13] Vaccarino V, Rathore SS, Wenger NK (2005). Sex and racial differences in the management of acute myocardial infarction, 1994 through 2002. *New England Journal of Medicine*.

[14] Ramsey DJ, Goff DC, Wear ML, Labarthe DR, Nichaman MZ (1997). Sex and ethnic differences in use of myocardial revascularization procedures in Mexican Americans and non-Hispanic whites: the Corpus Christi Heart Project. *Journal of Clinical Epidemiology*.

[15] Giacomini MK (1996). Gender and ethnic differences in hospital-based procedure utilization in California. *Archives of Internal Medicine*.

[16] Yarzebski J, Bujor CF, Lessard D, Gore JM, Goldberg RJ (2004). Recent and temporal trends (1975 to 1999) in the treatment, hospital, and long-term outcomes of Hispanic and non-Hispanic white patients hospitalized with acute myocardial infarction: a population-based perspective. *American Heart Journal*.

[17] Cohen MG, Roe MT, Mulgund J (2006). Clinical characteristics, process of care, and outcomes of Hispanic patients presenting with non-ST-segment elevation acute coronary syndromes: results from Can Rapid risk stratification of Unstable angina patients Suppress ADverse outcomes with Early implementation of the ACC/AHA Guidelines (CRUSADE). *American Heart Journal*.

[18] Marmot M (2005). Social determinants of health inequalities. *Lancet*.

[19] Mensah GA (2005). Eliminating disparities in cardiovascular health: six strategic imperatives and a framework for action. *Circulation*.

[20] Thygesen K, Alpert JS, White HD (2007). Universal definition of myocardial infarction. *Circulation*.

[21] Moore DS (2007). *The Basic Practice of Statistics*.

[22] Krumholz HM, Anderson JL, Bachelder BL (2008). ACC/AHA 2008 performance measures for adults with ST-elevation and non-ST-elevation myocardial infarction. *Journal of the American College of Cardiology*.

[23] Hasnain-Wynia R, Baker DW, Nerenz D (2007). Disparities in health care are driven by where minority patients seek care: examination of the hospital quality alliance measures. *Archives of Internal Medicine*.

[24] Echols MR, Mahaffey KW, Banerjee A (2007). Racial differences among high-risk patients presenting with non-ST-segment elevation acute coronary syndromes (results from the SYNERGY trial). *American Journal of Cardiology*.

[25] Hertz RP, Unger AN, Ferrario CM (2006). Diabetes, hypertension, and dyslipidemia in Mexican Americans and non-Hispanic whites. *American Journal of Preventive Medicine*.

[26] Winkleby MA, Kraemer HC, Ahn DK, Varady AN (1998). Ethnic and socioeconomic differences in cardiovascular disease risk factors: findings for women from the third national health and nutrition examination survey, 1988-1994. *Journal of the American Medical Association*.

[27] Cohen MG, Fonarow GC, Peterson ED (2010). Racial and ethnic differences in the treatment of acute myocardial infarction: findings from the get with the guidelines-coronary artery disease program. *Circulation*.

[28] Rodriguez F, Joynt KE, López L, Saldaña F, Jha AK (2011). Readmission rates for Hispanic Medicare beneficiaries with heart failure and acute myocardial infarction. *American Heart Journal*.

[29] Sada MJ, French WJ, Carlisle DM, Chandra NC, Gore JM, Rogers WJ (1998). Influence of payor on use of invasive cardiac procedures and patient outcome after myocardial infarction in the United States. *Journal of the American College of Cardiology*.

[30] Pilote L, Miller DP, Califf RM, Rao JS, Weaver WD, Topol EJ (1996). Determinants of the use of coronary angiography and revascularization after thrombolysis for acute myocardial infarction. *New England Journal of Medicine*.

[31] Ginzberg E (1991). Access to health care for Hispanics. *Journal of the American Medical Association*.

[32] Diez Roux AV (2005). Persistent social patterning of cardiovascular risk: rethinking the familiar. *Circulation*.

[33] Woodward M, Brindle P, Tunsfall-Pedoe H (2007). Adding social deprivation and family history to cardiovascular risk assessment: the ASSIGN score from the Scottish Heart Health Extended Cohort (SHHEC). *Heart*.

[34] Anderson LM, Scrimshaw SC, Fullilove MT, Fielding JE, Normand J (2003). Culturally competent healthcare systems: a systematic review. *American Journal of Preventive Medicine*.

[35] Zuvekas SH, Taliaferro GS (2003). Pathways to access: health insurance, the health care delivery system, and racial/ethnic disparities, 1996–1999. *Health Affairs*.

[36] Pérez-Stable EJ, Nápoles-Springer A, Miramontes JM (1997). The effects of ethnicity and language on medical outcomes of patients with hypertension or diabetes. *Medical Care*.

[37] Duran DG, Reyes C, Villarruel A (2001). *Quality Health Services for Hispanics*.

[38] U.S. Census Bureau Sex by occupation for the civilian employed population 16 years and over (Hispanic or Latino). www.census.gov/compendia/statab/2012/tables/12s0616.pdf.

[39] Diversity in the Physician Workforce: Facts & Figures 2006. www.aamc.org/factsandfigures.

[40] Jollis JG, Delong ER, Peterson ED (1996). Outcome of acute myocardial infarction according to the specialty of the admitting physician. *New England Journal of Medicine*.

